# Aeromonas nosocomial cluster: Investigation review of possible modes of transmission

**DOI:** 10.1017/ash.2023.357

**Published:** 2023-09-29

**Authors:** Anjali Bisht, Hannah Gray, JR Caldera, Shangxin Yang, Dan Uslan

## Abstract

**Background:** *Aeromonas* is a gram-negative rod known to be present in water, sewage and soil which may cause infections especially in immunocompromised hosts. Cases of *Aeromonas* gastroenteritis have been associated with warmer weather. In total, 3 patients with extensively drug resistant (XDR) Aeromonas were identified at our facility between August and September 2022 on 2 intensive care units (ICUs). Our infection prevention, microbiology, and facility teams investigated these cases to determine whether a common source could be the mode of transmission. **Methods:** To first determine whether patients’ *Aeromonas* specimens were related, whole-genome sequencing (WGS) of the clinical isolates from 3 patients was performed using the Illumina DNA Prep Kit and Illumina MiSeq. Sequencing analysis was performed using CLC Genomics Workbench for de novo assembly, single-nucleotide polymorphisms (SNP) calling, and tree generation, Geneious Prime for reference-based assembly, annotation, and quality assessment, KmerFinder for reference identification, and the Comprehensive Antibiotic Resistance Database for resistance gene detection via protein homology. Chart review revealed that patients occupied multiple rooms between 2 ICUs (Fig. 1). Because water is a known source of *Aeromonas*, facility records were reviewed for water intrusion events. This analysis identified several cases in the affected patient and surrounding rooms. Sinks and faucets from 10 rooms were swabbed followed by direct plating on blood, MacConkey agar, and *Aeromonas*-selective cefsulodin-Irgasan-novobiocin (CIN) agar plates. Lastly, the city temperatures before and after positive cases were reviewed to identify whether any correlation could be shown between temperature and timing of infection. **Results:** WGS analysis revealed that the 3 *Aeromonas* isolates (all identified as *A. hydrophila*) were not directly related (minimum distance, 934 SNPs) and harbored between 4 and 19 unique antimicrobial resistance genes, including co-occurring carbapenemases VIM-2 and KPC-3 in 1 isolate and OXA-232 in another. Of the 20 environmental samples, few gram-negative nonfermenting bacteria and no *Aeromonas* isolates were detected (Fig. 1). Elevated city did loosely proceed patient cases of *Aeromonas*, suggesting a possible role of higher temperature, which may have promoted the growth of *Aeromonas* during the periods of the 3 cases and thus may contribute to the nosocomial infections (Fig. 2). **Conclusions:** Although our investigation did not reveal a definitive cause for the *Aeromonas* cases, it did show the importance prompt identification and investigation can have on mitigating the spread of a cluster. Our facility has not identified any additional nosocomial cases. Monitoring water intrusion events and plans for remediation continue to be a priority.

**Figure:**
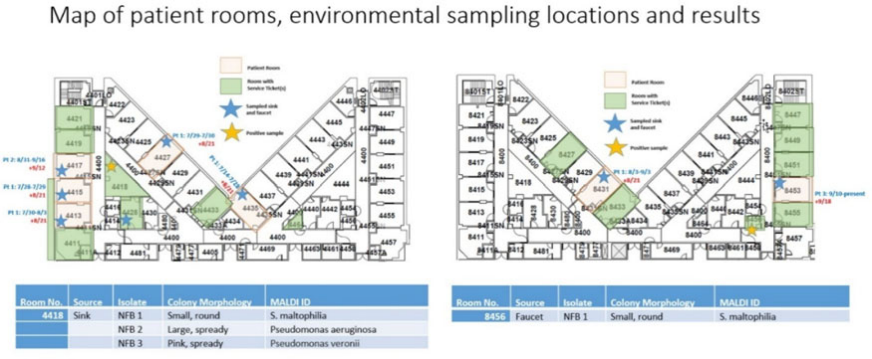

**Figure:**
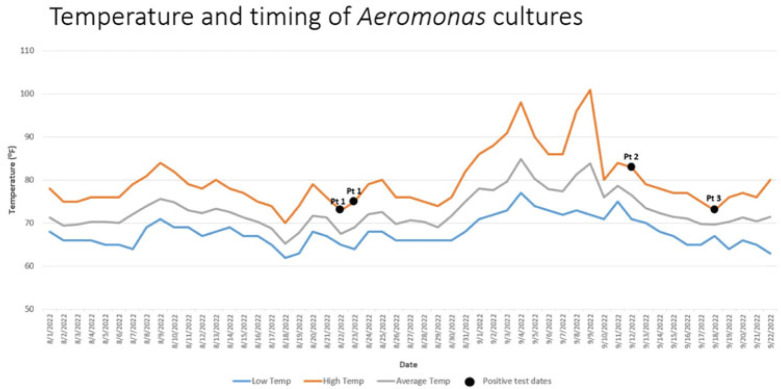

**Disclosures:** None

